# Sleep Deprivation in Young and Healthy Subjects Is More Sensitively Identified by Higher Frequencies of Electrodermal Activity than by Skin Conductance Level Evaluated in the Time Domain

**DOI:** 10.3389/fphys.2017.00409

**Published:** 2017-06-20

**Authors:** Hugo F. Posada-Quintero, Jeffrey B. Bolkhovsky, Natasa Reljin, Ki H. Chon

**Affiliations:** Biomedical Engineering Department, University of ConnecticutStorrs, CT, United States

**Keywords:** electrodermal activity, heart rate variability, autonomic nervous system, sleep deprivation, performance

## Abstract

We analyzed multiple measures of the autonomic nervous system (ANS) based on electrodermal activity (EDA) and heart rate variability (HRV) for young healthy subjects undergoing 24-h sleep deprivation. In this study, we have utilized the error awareness test (EAT) every 2 h (13 runs total), to evaluate the deterioration of performance. EAT consists of trials where the subject is presented words representing colors. Subjects are instructed to press a button (“Go” trials) or withhold the response if the word presented and the color of the word mismatch (“Stroop No-Go” trial), or the screen is repeated (“Repeat No-Go” trials). We measured subjects' (*N* = 10) reaction time to the “Go” trials, and accuracy to the “Stroop No-Go” and “Repeat No-Go” trials. Simultaneously, changes in EDA and HRV indices were evaluated. Furthermore, the relationship between reactiveness and vigilance measures and indices of sympathetic control based on HRV were analyzed. We found the performance improved to a stable level from 6 through 16 h of deprivation, with a subsequently sustained impairment after 18 h. Indices of higher frequencies of EDA related more to vigilance measures, whereas lower frequencies index (skin conductance leve, SCL) measured the reactiveness of the subject. We conclude that indices of EDA, including those of the higher frequencies, termed TVSymp, EDASymp, and NSSCRs, provide information to better understand the effect of sleep deprivation on subjects' autonomic response and performance.

## Introduction

This study analyzes the variations in electrodermal activity (EDA) and heart rate variability (HRV) measures during a 24-h period of sleep deprivation. Understanding the effects of sleep deprivation on human physiology is key to comprehending and mitigating the performance impairment produced by drowsiness. Human performance deterioration causes accidents in jobs like the military, transportation, health care, and many more that frequently require working long hours or late at night (Costa, 1996). Occupational accidents result in a large social and economic cost (Lyznicki et al., 1998; Leger, 1994).

Drowsiness has a remarkable effect on the autonomic nervous system (ANS) (Zhong et al., 2005; Michail et al., 2008; Liu et al., 2015). The ANS compensates for increased levels of stress by modulating the balance between parasympathetic and sympathetic nervous system traffic within the body's regulatory network. A predominantly sympathetic response to fatigue stressors is appropriate and beneficial. In contrast, a predominantly parasympathetic response to the same fatigue stressors reflects an inappropriate response, indicating a progression toward a state of decompensation and failure of physiological functions (Baharav et al., 1995; Winchell and Hoyt, 1997; Cooke et al., 2006; Furman et al., 2008; Michail et al., 2008). Given the high sensitivity of the ANS to drowsiness, the ANS is an attractive target for the development of an objective physiological measure of the effects of sleep deprivation.

Spectral analysis of HRV is a noninvasive way to quantitatively assess the dynamics of the ANS (Task Force of the European Society of Cardiology and the North American Society of Pacing and Electrophysiology, 1996). The high-frequency components of HRV (HRVHF, 0.15–0.4 Hz) are known to be solely influenced by the parasympathetic system; the low-frequency components (HRVLF, 0.045–0.15 Hz) are influenced by both the sympathetic and parasympathetic nervous systems. Despite the spectral overlap, HRVHF is widely used as a marker of parasympathetic function and HRVLF is used as a marker of sympathetic function (Task Force of the European Society of Cardiology and the North American Society of Pacing and Electrophysiology, 1996). Many studies have used indices of HRV to track the effects of sleep deprivation on humans (Nakano et al., 2000; Zhong et al., 2005; Viola et al., 2008; Pagani et al., 2009; Fogt et al., 2010, 2011; Chua et al., 2012; Glos et al., 2014; Vicente et al., 2016), with different levels of success. These studies suggest that HRV has the potential to track several effects of sleep deprivation.

Given the need to improve the assessment of ANS dynamics using noninvasive means, new methods for the signal processing of electrodermal activity (EDA) has gained popularity (Benedek and Kaernbach, 2010; Colbert et al., 2011; Boucsein et al., 2012; Bach and Friston, 2013; Freeman and Chapleau, 2013; Chaspari et al., 2015; Greco et al., 2016). The impetus for this innovation is that EDA reflects only activity within the sympathetic branch of the autonomic nervous system, as there is no parasympathetic innervation of eccrine sweat glands. EDA is a measure of the changes in electrical conductance of the skin, with strong correlation to sweat production, making it a pure assay of sympathetic activity (Dawson et al., 2007). EDA signals have been traditionally analyzed in the time domain looking at skin conductance level (SCL) and skin conductance responses (SCRs) (Boucsein et al., 2012). SCL accounts for the slow shifts and the SCRs are the rapid phase shifts of the EDA signal. Moreover, indices based on time-invariant and time-variant spectral analyses of EDA have been recently reported as measures of sympathetic control (Posada-Quintero et al., 2016a,b). Integrating frequency and time domains into the analysis of EDA has improved the consistency and sensitivity of the technique (Posada-Quintero et al., 2016b).

The effect of sleep deprivation on humans' EDA has been scarcely explored. However, recent studies suggest the important contribution of EDA and the sympathetic function assessment to better understanding the altered reactiveness of sleep-deprived people (Miró et al., 2002; Liu et al., 2015). Surprisingly, those studies only looked at the very slow components of EDA, i.e., change in SCL, and did not explore the occurrence of rapid shifts, which are common in the EDA signal. We hypothesize that higher-frequency components of EDA contain valuable information to better understand the effects of sleep deprivation. We expect the changes found in the EDA and HRV due to prolonged sleep deprivation can be used to prevent the inconvenient (many times fatal) consequences of sleep deprivation. To test this, we have employed a simple yet robust test that requires the subject to maintain vigilance. We have collected measurements of HRV and EDA through a 24-h sleep deprivation period, and analyzed the results.

## Materials and methods

### Subjects

Ten healthy volunteers (seven males and three females; ages ranging from 25 to 35) were enrolled in this study. Participants were asked to remain awake for at least 24 h, and various precautions were taken to ensure the validity of this study as well as the safety of the participants (e.g., experimenter observation). Seven days prior to the experiment, participants were given a data sheet to record their sleep patterns, to indicate compliance to the experimental constraints and expose potential outliers.

### Protocol

Participants were asked to halt all consumption of stimulants and depressants beginning 48 h prior to the start of the experiment. Food was provided to ensure it adhered to the restrictions of this study. Within 2 h of waking up on the morning the study started, participants arrived at the experimental facility located at the University of Connecticut. Participants remained in the building for approximately 25 h to allow for the completion of all trials. Within the first hour of arrival, participants completed a set of learning trials for the experimental task. They then proceeded to perform the task every other hour for the duration of the 24-h period.

We have called a “run” to be each of the 13 experimental sessions. Run 1 happened right after the subject arrived at the experimental location and the test was explained. Run 25 occurred after 24 h of sleep deprivation. Five minutes before each task run, stainless steel electrodes were placed on the middle and index fingers of each participant's non-dominant hand to collect EDA signals. A 5-min of rest was allowed, to procure hemodynamic stabilization. A set of three standard Ag/AgCl electrodes were placed on each participant's chest for recording ECG. ECG and EDA signals were simultaneously recorded every session, at a sampling rate of 400 Hz. An HP 78354A ECG monitor (Hewlett-Packard, FDA approved) and a GSR amplifier FE116 (fully isolated AC excitation and automatic zeroing low voltage amplifier, 22 mVrms @75 Hz, ADINSTRUMENTS) were used to collect ECG and EDA, respectively. No on-line filtering was applied during the signal recording. The EDA device was adjusted to zero at the start of every run to assure a consistent level and to avoid baseline differences in EDA that might have arisen from homeostatic and circadian processes (Miró et al., 2002; Miller and Gronfier, 2006), allowing changes specific to the test to be isolated.

Between runs, leads were disconnected. During this period subjects were instructed to remain in the building, and were allowed to eat (food was provided to ensure adherence to the protocol) or do any activity other than exercising or sleeping (reading, watching movies, going to the restroom, talking on the phone, etc.), to avoid any undesirable influence in sympathetic arousal. The same instructions were followed during the day and the night. We did not video record the test; however, an experimenter was with the subject all the time. The study protocol was approved by the Institutional Review Board of The University of Connecticut and all volunteers consented to be subjects for the experiment. Data collected in this work can be accessible to interested researchers. Please contact the corresponding author.

#### Error awareness task

**Figure 1:**
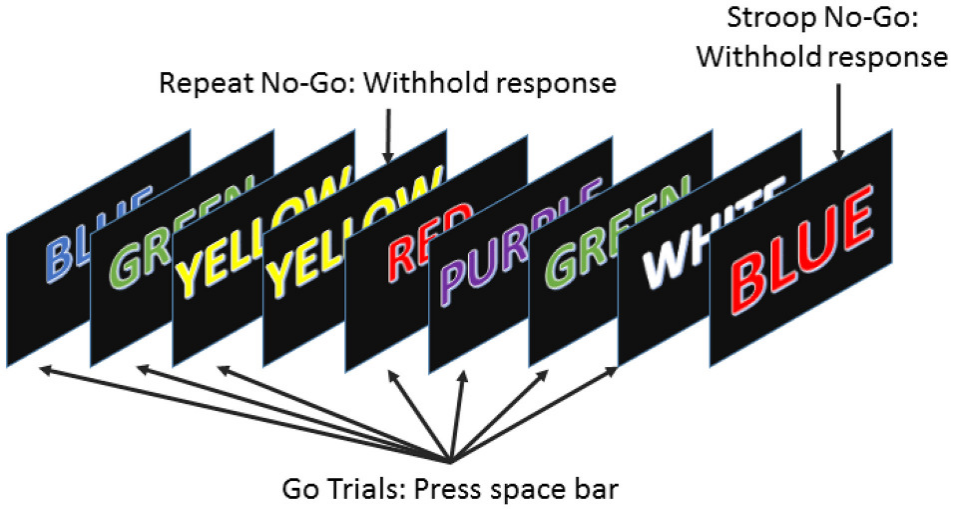
The error awareness task required subjects to respond to a stream of color words by pressing the space bar, and to withhold their response when either a word was repeated on consecutive trials or the color and word were incongruous.

To examine the effect of sleep deprivation on subjects' performance, we used the error awareness task (EAT). The EAT involves presenting a serial stream of single color words in fonts matching the color of the word, where each word is presented for 900 ms followed by a 600 ms inter-stimulus interval. In this study, EAT was 5 min long. Participants were trained to respond to each of the words with a single button press for “Go” trials and withhold this response (“No go” trials) when either of two different circumstances arose. The first withhold condition occurred if the same word was presented on two consecutive trials, while the second was if the word and color of the word did not match (see Figure 1; Hester et al., 2005). Notice that the latter situation presents a Stroop effect (but using a keyboard in lieu of the traditional verbal response to it), which induces cognitive stress in the subjects (Stroop, 1935). The two withhold conditions forced subjects to be attentive rather than just repetitive, because the overlearned human behavior of reading disadvantages the color recognition. Being forced to also monitor for the color match kept the subjects as alert as they could be. We computed the “Go” trials response time for every trial, as a measure of each subject's performance, to be able to examine the effects of sleep deprivation on such a simple measure and correlate it with the various available physiological parameters.

### Physiological indices of the autonomic nervous system

Measures to assess the autonomic nervous system (ANS) based on analysis of HRV and EDA were computed using data collected while the subject was performing the EAT.

#### Indices of heart rate variability

For HRV analysis, ECG signals were band-pass filtered (0.05–40 Hz) to reduce noise and motion artifacts. The R-waveform peaks were detected using the detection algorithm that defines a delineation function based on the envelope of the ECG signal (Nygårds and Sörnmo, 1983; Vidaurre et al., 2011). All the segments were visually inspected to ensure that no beat was missed. After accounting for the missed R-wave beats, the HR time series were computed. The power spectra of HRV were then calculated using Welch's periodogram method with 50% data overlap. The RR interval series were converted to an evenly time-sampled signal (4 Hz) by cubic spline interpolation. A Blackman window (length of 256 points) was applied to each segment and the fast Fourier transform was calculated for each windowed segment. Finally, the power spectra of the segments were averaged. The low-frequency index (HRVLF [ms^2^], 0.045–0.15 Hz), high-frequency index (HRVHF [ms^2^], 0.15–0.4 Hz), and normalized versions of these two (HRVLFn, HRVHFn, dividing by total power of HRV, in normalized units [n.u.]) were computed (Task Force of the European Society of Cardiology and the North American Society of Pacing and Electrophysiology, 1996).

Four minutes of clean ECG signals (subjects were asked to remain still) were used to compute HRV indices. R-peak detection on ECG segments was visually inspected to eliminate any ectopic beats. Indices from the LF range (HRVLF and HRVLFn) of HRV were used as indices of sympathetic control although they have proven parasympathetic influence, and indices from the HF power (HRVHF and HRVHFn) were used as indices of parasympathetic control, even though parasympathetic tone is present in both the LF and the HF range.

#### Indices of electrodermal activity

Indices of EDA were computed in the time and frequency domains. In the time domain, the EDA signal is traditionally decomposed into two measures: skin conductance level (SCL) and skin conductance responses (SCRs) (Boucsein et al., 2012). To extract the tonic component of the EDA signals, a 10th-order low-pass finite impulse response filter with a cut-off frequency of 0.0004 Hz was applied. We used a cutoff frequency of 0.0004 Hz in order to retain most of the signal dynamics except for the DC components below this cutoff frequency. Although the skin conductance level conceptually refers to the tonic EDA, the index of SCL is computed as the mean value of the tonic EDA taken during a 2-minute period. The remaining signal (raw signal minus tonic component) was used to compute the phasic component, which was used to count the SCRs. The SCRs are the rapid transient events contained in the EDA signals. The index of non-specific SCRs (NSSCRs) was obtained manually for each minute and then averaged over the 2-min period. It is important to note that a minimum change in conductance needs to be considered for defining non-negligible SCRs. The recommended threshold value of 0.05 μS was used (Boucsein et al., 2012). In addition, when a second response occurred before completion of the prior response, the two responses were counted as two positive NS.SCRs even though they overlapped. There are also publicly-available toolboxes for computing the above-mentioned indices (e.g., pspm.sourceforge.net and www.ledalab.de). In this study, for each run, 2 min of EDA were collected while the subject was performing the EAT, to compute SCL and NSSCRs.

The same 2 min of EDA extracted to compute time-domain indices were also used for spectral indices. EDA was down sampled to 2 Hz prior to spectral analysis. The time-invariant spectra of EDA were calculated using Welch's periodogram method with 50% data overlap. A Blackman window (length of 128 points) was applied to each segment, the fast Fourier transform was calculated for each windowed segment, and the power spectra of the segments were averaged. The power spectral index of EDA, EDASymp [μS^2^], was computed by integrating the power in the range from 0.045 to 0.25 Hz, as it was found before to be sensitive to cognitive stress (Posada-Quintero et al., 2016a).

To compute the time-varying index of EDA, TVSymp, the time-frequency representation (TFR) of EDA was computed using the variable frequency complex demodulation (VFCDM), a time-frequency spectral (TFS) analysis technique that provides accurate amplitude estimates and one of the highest time-frequency resolutions (Chon et al., 2009). The components comprising the frequency power in the range from 0.08 to 0.24 Hz were used to compute TVSymp.

### Statistics

The set of physiological indices tested in this study included: HRVLF, HRVLFn, HRVHF, HRVHFn, SCL, NSSCRs, EDASymp, and TVSymp. As a measure of subjects' performance, average “Go trial” reaction time (Go_RT), Stroop No-Go and Repeat No-Go accuracies (Stroop_NoGo_acc and Repeat_NoGo_acc, respectively) were computed for every run of every subject. Go_RT exhibits an inverse relationship to subjects' performance, as a higher reaction time represents lower performance. We explored the differences caused by the sleep deprivation on the physiological indices.

#### Changes on indices due to sleep deprivation

The impairment of participants' performance due to sleep deprivation was tested, after which all the computed physiological indices of ANS were analyzed to test for significant differences due to sleep deprivation (*p* < 0.05). In both cases (performance and physiological indices) we used the Friedman test, a non-parametric statistical test similar to the parametric repeated measures ANOVA, used for one-way repeated measures analysis of variance by ranks (Friedman, 1937). We used the resulting average ranks of the Friedman test for each index, to perform multiple comparisons and determine significant differences between runs. The analysis is meant to test the relationship between performance deterioration and changes to physiological indices, allowing us to understand the effect of prolonged wakefulness on subjects' EDA.

## Results

**Figure 2:**
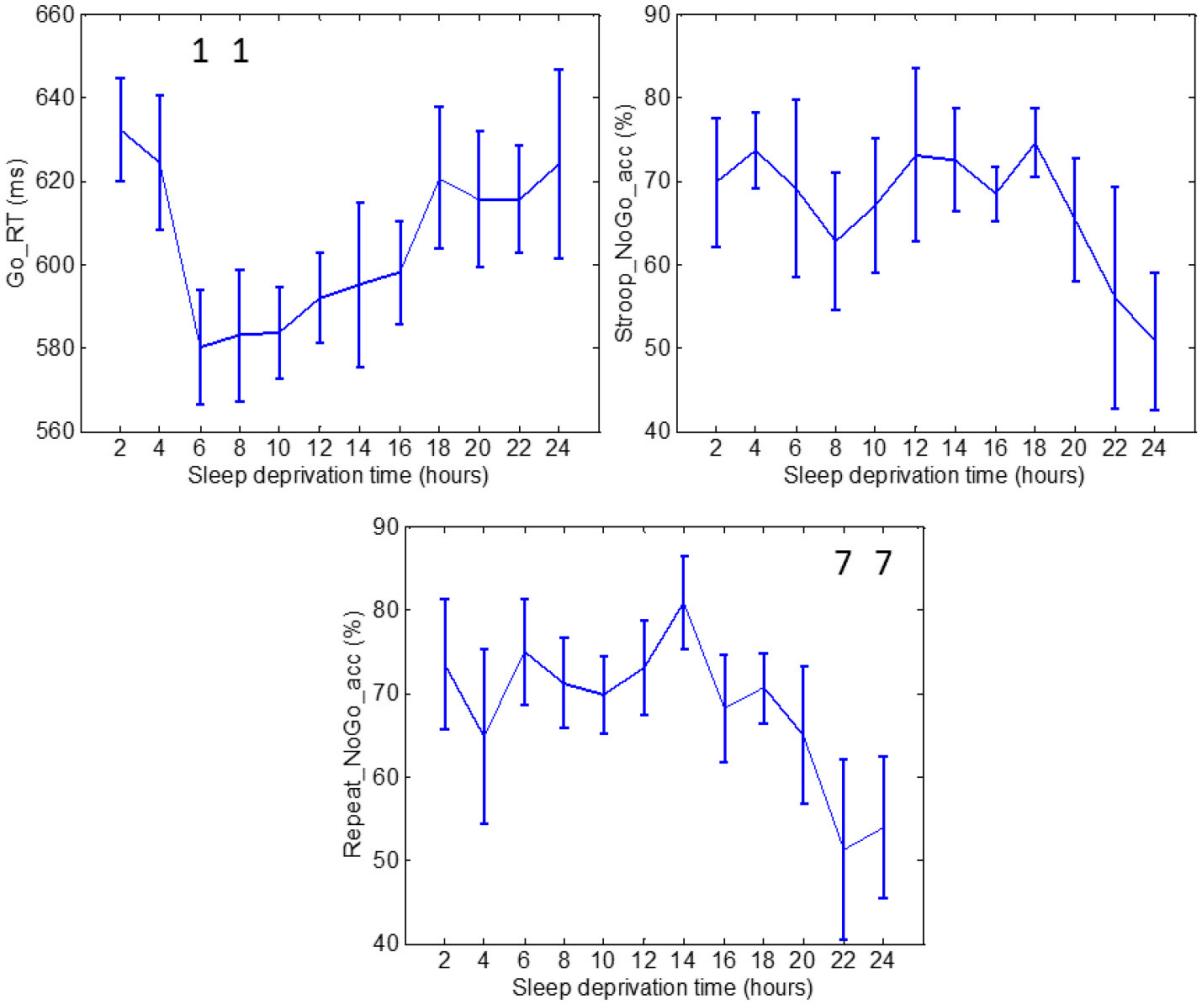
Go_RT, Stroop_NoGo_acc and Repeat_NoGo_acc values through 24 h of sleep deprivation. Numbers on the figure represent the runs it was significantly different to. Whiskers account for one standard deviation.

**Figure 3:**
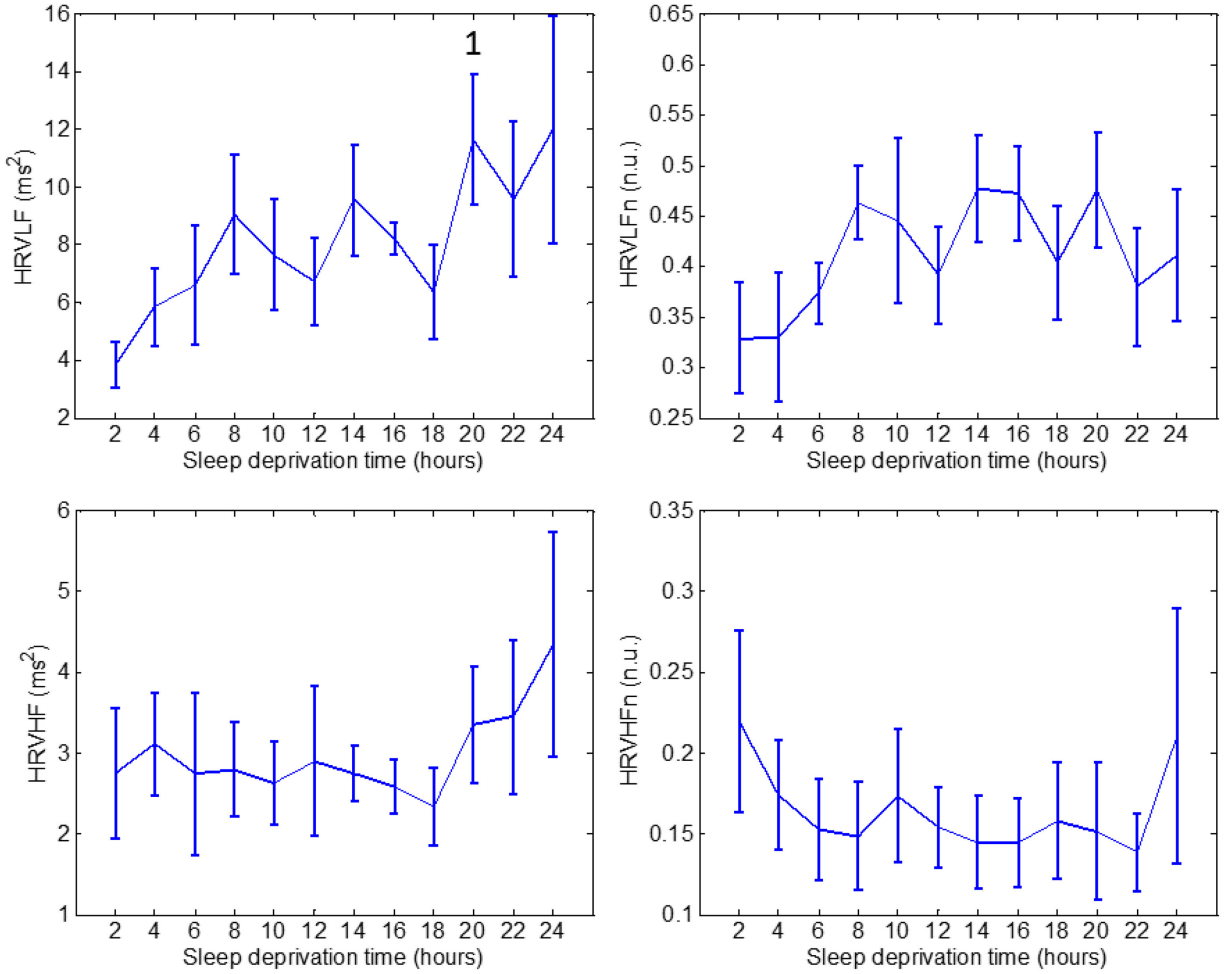
Values through 24 h of sleep deprivation for HRV indices: HRVLF **(top left)**, HRVLFn **(top right)**, HRVHF **(bottom left)** and HRVHFn **(bottom right)**. Numbers on the figure represent the runs it was significantly different to. Whiskers account for one standard deviation.

**Figure 4:**
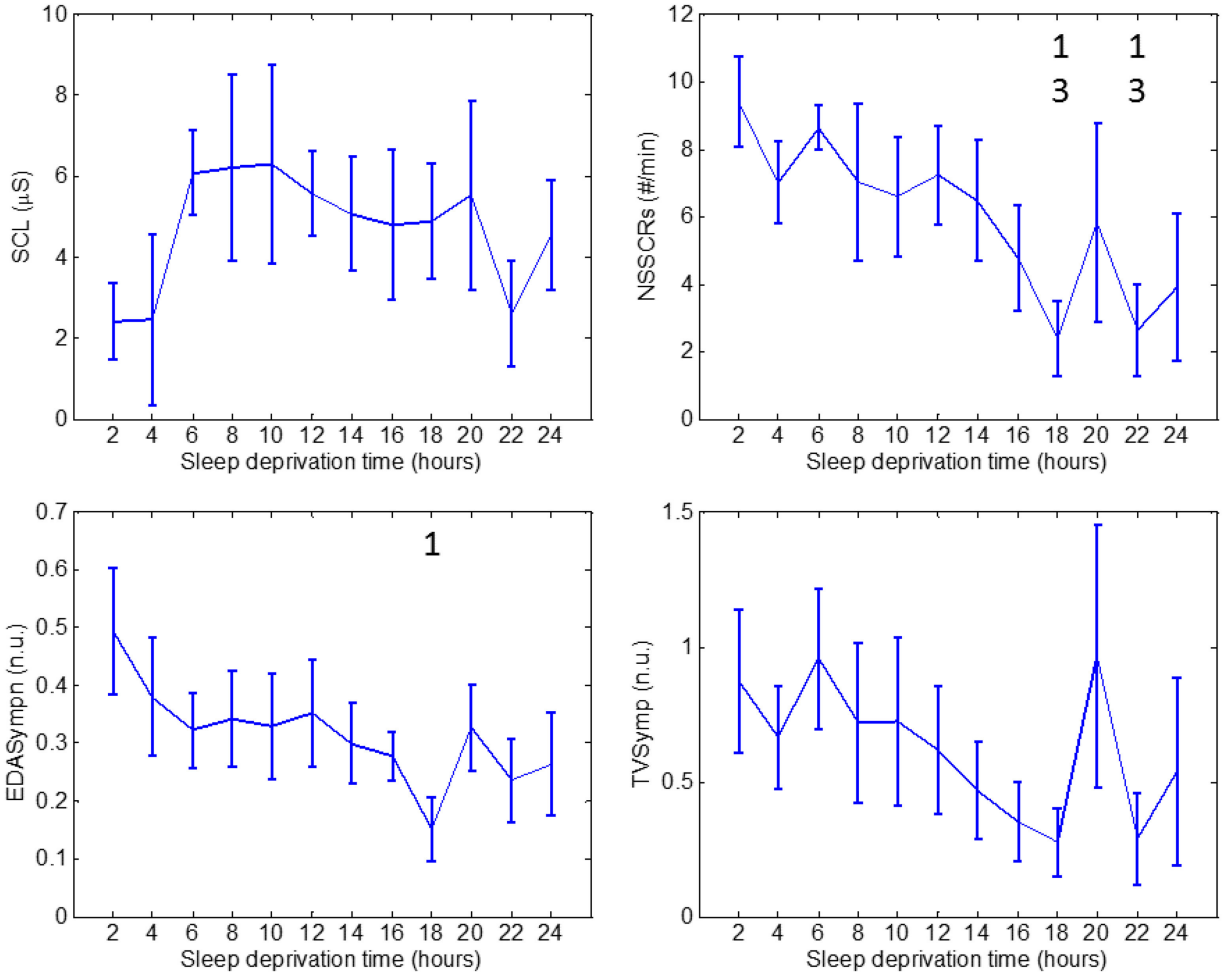
Values through 24 h of sleep deprivation for EDA indices: SCL **(top left)**, NSSCRs **(top right)**, EDASymp **(bottom left)** and TVSymp **(bottom right)**. Numbers on the figure represent the runs it was significantly different to. Whiskers account for one standard deviation.

For clarity and simplicity, we will refer to the run number to express the results, and the reader can easily abstract the relationship between run number and hours of sleep deprivation (hours of deprivation = 2^*^ run number). The changes in performance and ANS indices as a result of sleep deprivation are shown on Figures 2–4. The figures display the results for Go_RT, HRV and EDA indices, respectively. Using the Friedman test, we found significant effects of sleep deprivation in Go_RT, Repeat_NoGo_acc, HRVLF, NSSCRs, and TVSymp (*p* < 0.05). For comparison between runs, significant differences are marked in the figures.

Measures of Go_RT during runs 3 and 4 were significantly different to the measurement during run 1. Measurements of Go_RT during runs 6 and 7 exhibited significantly lower values than those in run 2. The average reaction time increased after runs 6 and 7, but the increase was not significant. The results of Go_RT can be described in the following manner: the first two runs constituted a learning period (2 h) after which the subjects reached a plateau where the average reaction time stabilized for runs 3 through 8, ending in a final increase in average reaction time. Stroop_NoGo_acc and Repeat_NoGo_acc indices exhibited stable values for most of the test, followed by a sustained drop in value after 18 h. Repeat_NoGo_acc showed a significant decrease in runs 11 and 12 compared to run 7; in this run Repeat_NoGo_acc exhibited a peak value. Differences in Stroop_NoGo_acc were not significant.

HRVLF exhibited a significant increase in run 10, compared to run 1. None of the other HRV indices: HRVLFn, HRVHF, or HRVHFn, showed a significant difference during the 24-h period.

As for EDA measures, we found no significant differences in SCL values. SCL profile looks similar to Go_RT for runs 1 through 8, in the sense that there are two runs similar to learning, followed by a plateau. In fact, the correlation between SCL and Go_RT is −0.82. The NSSCRs were significantly diminished on runs 9 and 11 compared to runs 1 and 3. Values of the normalized EDASymp (EDASympn) were different at run 9 compared to run 1. TVSymp, similarly to NSSCRs and EDAsympn, exhibited a continuous decrease in magnitude over time. However, that decrease was not significant for TVSymp at any time.

## Discussion

We have observed that indices representing higher frequencies of EDA follow a trend similar to indices of vigilance, measured by accuracy in performing an attention task, whereas the index that represents the slow changes of EDA resembles more the changes in reactiveness, measured by reaction time. This observation was also found in a previous study (Miró et al., 2002). Our results suggest that indices of EDA can complement HRV measures, to predict the effect of sleep deprivation on humans' autonomic response and performance.

Many projects have studied the ANS using HRV under sleep deprivation. A group studied participants during 40 h of constant routine conditions and determined that HRV was in general diminished during recovery sleep after sleep deprivation (Viola et al., 2008). Analysis of these results revealed a decrease in HRV due to sleep deprivation, but with less intensity than during sleep. Another group found, in persons with over 36 h of sleep deprivation, a progressive increase during daytime in sympathetic tone, and a decrease in parasympathetic cardiac autonomic modulation (Zhong et al., 2005). This was demonstrated by reduced HRV values in the frequency domain for healthy participants performing a cognitive task repetitively. In contrast, another study could not find increased sympathetic drive expressed during daytime by diminished HRV values after one night of sleep deprivation (Pagani et al., 2009). Another investigation revealed a correlation of HRV spectral values with subjects' performance in a psychomotor vigilance test during sleep deprivation (Chua et al., 2012).

Information on the effect of sleep deprivation on humans' electrodermal activity is scarce. A classical review reported contradictory results on changes in SCL and SCRs during sleep deprivation (Horne, 1978). However, that review highlighted that many of these studies suffered from methodological limitations (single case studies, measurements taken only once or twice during the test, or lack of statistical analysis). A study later reported an increased latency and reduced amplitude in event-related SCRs after 36 h of sleep deprivation (McCarthy and Waters, 1997). There are very few recent investigations. A study reported an increase of skin resistance level (inverse to SCL) that correlated with deterioration of reaction time, using a simple reaction time test (Miró et al., 2002). Indeed, this is what we observe with our data. We found a high negative correlation value of −0.82 between the mean values of SCL and Go_RT. A recent study looked at the SCL to probe how the sympathetic nervous system might contribute to altered reactiveness in sleep deprived persons (Liu et al., 2015). Note that these two recent studies only analyzed the SCL, not including the information of the more rapid transients of EDA, where this signal has shown great sensitivity.

We found a significant reduction in Go_RT in runs 3 and 4 compared to run 1 (a learning effect), and an increase after about 16 h of sleep deprivation. Stroop_NoGo_acc and Repeat_NoGo_acc (indices of vigilance) were decreased toward the end of the 24-h period. These results were expected, as the human brain is able to maintain stable cognitive performance over some period of time, but after prolonged wakefulness the level of arousal and performance tends to diminish (Lorenzo et al., 1995; Porkka-Heiskanen et al., 1997; Franco et al., 2004; Volkow et al., 2008; Kaplan et al., 2017; Legault et al., 2017). In agreement to the affected levels of arousal, indices of sympathetic control should ideally exhibit a decreasing trend. In this study, values of HRV indices of sympathetic control steadily increased with sleep deprivation (with some fluctuations), whereas NSSCRs, EDASymp and TVSymp tended to lower values by the end of the test.

EDAsymp exhibited a significant decrease in run 9 with respect to run 1, which coincided with an increase in mean Go_RT, and preceded the decrease in mean Stroop_NoGo_acc and Repeat_NoGo_acc noticeable in run 10. A similar peak in run 9 was also detected by the NSSCRs index, with a repeated peak in run 11. The profiles of NSSCRs, EDASympn and TVSymp were similar, although EDASympn was somewhat smoother. In terms of significant change, it is noticeable that indices of higher-frequency components of EDA provide more information to understand and possibly predict the effects of sleep deprivation on the sympathetic nervous system.

There is an apparent relationship between the performance indices (Go_RT, Stroop_NoGo_acc and Repeat_NoGo_acc) and the indices of EDA. First, Go_RT and SCL show a remarkable inverse relationship (correlation coefficient value of −0.82), showing a training period (the first two runs), a stable plateau for a long part of the test (top performance, after subjects learned the task), and a period when the indices went back close to training values. We hypothesize that this final period, characterized by a drop in reactiveness (higher reaction time), is due to fatigue induced by sleep deprivation. This means that tonic SCL relates to subjects' reactiveness, which is in agreement with previous studies (Hochberg et al., 1971; Miró et al., 2002; Liu et al., 2015).

Subjects' performance measured by reaction time indices and EDA during periods of sleep deprivation is sensitive to both circadian rhythms and skin sympathetic arousal. However, it is known that sleep deprivation causes an overall slowing of reaction times and increased errors of omission and commission (Lim and Dinges, 2008). Indeed, this is the effect we observe on the measures of performance used in this study, as there is a trend in the increase in reaction time and a decrease in accuracy toward the end of the 24-h period (see top panels of Figure 2). The first data points in the top panels of Figure 2 were collected at 10 AM and the last 3 data points were taken at 4, 6 and 8 AM. At 4, 6, and 8 AM, the circadian rhythm would be in play and would cause the Go_RT reaction time to increase. However, instead we observe in the top panels of Figure 2 a continued trend of increase in the reaction time and decrease in accuracy. Hence, the trend of increase in reaction time with concomitant increase in sleep deprivation, shown in Figure 2, indicates that subjects are more affected by sleep deprivation than by the circadian rhythm. Similar results can be seen in Figure 4, with the same interpretation. It should be noted that we do observe a single value increase in the EDA indices in the panels of Figure 4 at the 20th hour (4 a.m.) which is likely due to circadian effect.

Second, indices that account for higher frequencies, NSSCRs, EDASymp, and TVSymp, relate to the measures of No-Go accuracy. These measures, Stroop_NoGo_acc and Repeat_NoGo_acc, which represent vigilance, remained stable for a part of the test (5–6 runs) then constantly degraded to low values. NSSCRs, EDASymp and TVsymp exhibited behavior similar to the indices of vigilance, but at 20 h they presented a large overshoot, followed by a final return to low values. We believe that this high peak toward the end of the test suggests that at that time the subjects made an attempt to stay focused and vigilant. However, improved accuracy is not achieved. The results are in agreement with the classic studies that reported a decrease in amplitude of SCRs after sleep deprivation (McCarthy and Waters, 1997), and with the classic report that related SCRs to attention, noting that such responses are sensitive to stimulus novelty (Hochberg et al., 1971).

The only available indices of parasympathetic control, HRVHF and HRVHFn indices, were stable throughout the test, with an increase in mean value toward the end of the 24 h. These indices, especially HRVHFn, resembles the sleepiness of subjects at the start of the test, and the increased sleepiness by the end of the study.

### Limitations

In this study, only 10 young and healthy subjects were studied. This is a 25-h sleep deprivation study with quite demanding study protocols. It was difficult to obtain subjects who were willing to participate in the study since it required sleep deprivation for 25 h. Moreover, it also required sleep deprivation for the conductors of the study, hence, it was a very demanding study from both the conductors' and subjects' perspectives. Given the limited sample size and characteristics of the set of subjects, observations are limited to a very specific group of individuals and more data are needed to draw general conclusions. Analysis on gender differences is even more limited with the current data set, and needs to be explored in future studies. Also, EAT provides a set of measures that cannot be considered a full assessment of performance. The indices are certainly linked to reactiveness and attention, but do not fully describe the kinds of tasks a subject encounters in real life. It should be noted that despite the unavoidable variability between subjects' skills and training conditions, and a limited sample size, we found considerable consistency in measures of performance throughout the ten subjects.

## Conclusion

We evaluated the effects of sleep deprivation on reactiveness, vigilance, HRV, and EDA, every 2 h during a 24-h period. We found notable differences in the effects of sleep deprivation on lower and higher frequencies of EDA. Information confined to the higher frequency components of EDA relates to subjects' level of attention or vigilance. For its part, changes in tonic SCL exhibited a relationship with subjects' reactiveness. As noted in the Limitation section, due to a limited sample size, we found only a few significant differences among runs for the indices examined in this work. However, there is a clear trend of inverse relationship between increases in reaction time (top left panel of Figure 2) and decreases in NSSCR, EDASympn and TVSymp indices as shown in Figure 4. Conversely, decreases in accuracy associated with Stroop NoGo and Repeat NoGo are highly correlated to similar decreases in NSCCR, EDASympn, and TVSymp. Thus, this information can potentially be used for developing tools to prevent the effects of prolonged wakefulness in jobs that require a high number of work hours along with significant concentration to perform well, to assess and predict impaired cognitive performance.

## Ethics statement

The study protocol was approved by the Institutional Review Board of The University of Connecticut and all volunteers consented to be subjects for the experiment. All subjects gave written informed consent in accordance with the Declaration of Helsinki.

## Author contributions

HP: performed experiments, data analysis and wrote a draft of the paper; JB: performed experiments and edited the paper. NR: performed experiments and edited the paper. KC: conceptualized the study and edited the final version of the paper.

### Conflict of interest statement

The authors declare that the research was conducted in the absence of any commercial or financial relationships that could be construed as a potential conflict of interest.

## Abbreviations

ANS: autonomic nervous system
EDA: Electrodermal Activity
HRV: heart rate variability
HRVLF: low frequency component of heart rate variability
HRVHF: high frequency component of heart rate variability
NSSCRs: non-specific skin conductance responses
RT: reaction time
SCL: skin conductance level.
